# Elevated TRIP13 drives the AKT/mTOR pathway to induce the progression of hepatocellular carcinoma via interacting with ACTN4

**DOI:** 10.1186/s13046-019-1401-y

**Published:** 2019-09-18

**Authors:** Meng-Xuan Zhu, Chuan-Yuan Wei, Peng-Fei Zhang, Dong-Mei Gao, Jie Chen, Yan Zhao, Shuang-Shuang Dong, Bin-Bin Liu

**Affiliations:** 1 Liver Cancer Institute, Zhongshan Hospital, Fudan University and Key Laboratory of Carcinogenesis and Cancer Invasion, Ministry of Education, 180 Fenglin Road, Shanghai, 200032 China; 2 Department of Liver Surgery, Liver Cancer Institute, Zhongshan Hospital, Fudan University, 180 FengLin Road, Shanghai, 200032 China

**Keywords:** TRIP13, HCC, miR-192-5p, ACTN4, EMT

## Abstract

**Background:**

ATPase associated with a variety of cellular activities (AAA ATPase) family members are closely linked to tumor formation and progression. However, their roles in hepatocellular carcinoma (HCC) largely remain unclear.

**Methods:**

Bioinformatic analyses of public databases were used to excavate the potential AAA ATPases that may contribute to HCC, and thyroid hormone receptor interactor 13 (TRIP13) was selected to following researches because of its most prominently differential expression. Western blot, qRT-PCR and immunohistochemistry were used to detect the expression of TRIP13 in HCC tissues, and then the relationship between TRIP13 expression and clinicopathological parameters were evaluated. Finally, its functions and potential mechanisms were investigated through a series gain- and loss-of-function strategies both in vitro and in vivo.

**Results:**

TRIP13 was significantly overexpressed in HCC tissues and high level of TRIP13 was closely correlated with a worse clinical outcome. Functionally, elevated TRIP13 facilitated cell proliferation, migration, invasion, and promoted cellular epithelial–mesenchymal transition (EMT) in vitro, while promote tumor growth and lung metastasis in vivo. Mechanistically, TRIP13 interacted with ACTN4 and positively regulated its expression, thus activating the AKT/mTOR pathway to drive tumor progression. Moreover, miR-192-5p served as an upstream regulator of TRIP13 by directly binding to TRIP13 mRNA 3′ UTR, which may partially explain the high expression of TRIP13 in HCC.

**Conclusion:**

Our findings identified TRIP13 as a promising candidate oncogene in HCC, and TRIP13 induced cell migration, invasion and metastasis of HCC through the AKT/mTOR signaling via interacting with ACTN4.

**Electronic supplementary material:**

The online version of this article (10.1186/s13046-019-1401-y) contains supplementary material, which is available to authorized users.

## Background

Hepatocellular carcinoma (HCC), accounting for more than 90% of liver cancers, is the fifth most common cancer and the third cause of cancer death worldwide. Despite feasible and effective treatment approaches such as surgical resection, orthotopic liver transplantation, and radiofrequency thermal ablation, the long-term prognosis for HCC patients remains unsatisfactory due to its aggressiveness and high recurrence rate after resection [1, 2]. Therefore, exploring the detailed mechanism underlying HCC progression and identifying novel therapeutic targets for treatment are especially urgent.

AAA ATPase family consists of 53 members, which are characterized by the presence of a highly conserved ATPase domain. By hydrolyzing ATP, proteins of this family generate mechanical energy to conformationally remodel proteins or polynucleotides, and thus playing critical roles in protein degradation, DNA replication and membrane fusion events [3]. Over the last few years, accumulated evidence has indicated that AAA ATPase family proteins participate in the development and progression of multiple tumors. For instance, Mikesch et al. reported that the AAA ATPase Reptin interacts with HDAC1 to drive tumor progression and resistance to cisplatin in non-small cell lung cancer [4]. Additionally, AAA ATPase p97, which plays a critical role in the regulation of protein homeostasis pathways, has been identified as a therapeutic target in ovarian cancer and multiple myeloma, and its inhibitor can effectively induce cancer cell death in vitro and inhibit tumor growth in vivo [5–7]. However, to date, little research has focused on the roles of AAA ATPase proteins in HCC pathogenesis.

In this study, by reanalyzing AAA ATPase gene expression profiles in the The Cancer Genome Atlas Liver Hepatocellular Carcinoma (TCGA-LIHC) data collection, we first identified TRIP13 was the most significant differential expressed gene. In recent years, the role of AAA ATPase TRIP13 in the occurrence and development progress of malignant tumors has received significant attention. Therefore, we subsequently examined TRIP13 expression and clinical significance in HCC samples, and performed loss- and gain-of-function assays to investigate its effects on HCC growth and metastasis in vitro and in vivo. Furthermore, we particularly demonstrated the underlying mechanism, through which TRIP13 is aberrantly overexpressed and induces the EMT in HCC cells.

## Materials and methods

### Bioinformatic analyses

The clinical information and RNAseqV2 data of 369 HCC patients were downloaded from the TCGA database using the GDC Data Portal (https://gdc-portal.nci.nih.gov). R software version 3.6.1 (https://www.r-project.org/) was used to identify differently express genes (DEGs) between HCC and normal tissues with the cut-off criteria of *P* < 0.00001 and log_2_ FC (fold-change) > 1.5. Kaplan-Meier analysis of 6 significantly upregulated AAA ATPase genes (TRIP13, CHTF18, RFC4, ATAD2, FIGL2, ATAD5) were performed by SPSS version 20.0 (SPSS, USA). mRNA expression of TRIP13 in Gene Expression Omnibus (GEO) datasets including GSE14520, GSE6764, and GSE3500 were collected from the Oncomine database (https://www.oncomine.org/resource/main.html).

### Patient samples

A total of 96 pairs of HCC and peritumor samples were obtained from patients who underwent initial surgical resection from January 2006 and December 2008 at Zhongshan Hospital Fudan University, and were made into tissue microarrays as previously described [8]. Another cohort of 20 fresh HCC cases were collected from the Department of Liver Surgery in Zhongshan Hospital. All the patients were confirmed as HCC independently by two pathologists. This study has been approved by the Institutional Review Board of Zhongshan Hospital Fudan University, and informed consent has been obtained from each patient.

### Immunohistochemistry

Immunohistochemistry were performed as our previous study [9]. Briefly, tumor tissue slides were rehydrated, antigen retrieved, blocked and incubated with primary antibodies. After 6 h, slides were treated with secondary antibodies and colored by DAB peroxidase substrate kit for IHC (Yeasen, China). The staining levels were quantitated with Image-Pro Plus 7.0 (Media Cybernetics, USA) as previously described [10]. In brief, three representative areas of each patient tissue were photographed (200×) and their density of positive staining were counted with the integrated absorbance and image area. Patients were divided into either high or low expression group by the average density.

### Cell lines and culture

Huh7, SMMC7721, and PLC cell lines were purchased from the Chinese Academy of Science Cell Bank (Shanghai, China). MHCC97H, MHCC97L, HCCLM3 cell lines were obtained from the Liver Cancer Institute of Zhongshan Hospital (Shanghai, China). All cell lines were cultured in DMEM (Hyclone, USA) supplemented with 10% fetal bovine serum at 37 °C in a humidified incubator with 5% CO_2_.

### Plasmids, lentiviral and RNA oligonucleotides

To knock down TRIP13, two different oligonucleotides sequences specific for TRIP13 were designed and constructed as the pLKO-shTRIP13 lentivirus. Additionally, TRIP13 cDNA (transcript variant 1) was cloned into lentiviral expression plasmid pLV5-GFP-Puro and constructed as pLV5-TRIP13 lentivirus. HCC cells were infected with lentivirus using polybrene (5 μg/ml) and subsequently selected with puromycin (5 μg/ml) for 3 days to establish the stable TRIP13-silencing or TRIP13-overexpressing cell lines. The siACTN4, siNC, miR-192-5p mimics, miR-192-5p inhibitor and miR-control were purchased from GenePharma (Shanghai, China), and transfected into cells using Lipofectamine 2000 ((Invitrogen, USA) according to the instructions.

### Real-time PCR and western blot analysis

Total RNA was purified using Mini BEST Universal RNA extraction KIT (TaKaRa) and cDNA was synthesized using the Prime-Script RT Master Mix (TaKaRa, Japan) according to the manufacturer’s instructions. qRT-PCR was performed using SYBR Green Realtime PCR Master Mix (Yeasen, China) in an ABI Prism 7500 system. Samples from each experiment were analyzed in triplicate. The primer sequences used in this study were as follows:

- sense: 5′-AAAATCTAGAATGGACGAGGCCGTGG3′,
- antisense: 5′-AAAAGGATCCTCAGATGTAAGCTGCAAGC-3′.

Western blot analysis was performed as in our earlier study [11]. Primary antibodies used in this study were as follows: anti-TRIP13 (Abcam, ab128171), anti-ACTN4 (CST, 15145), anti-GAPDH (Abcam, ab37168)), anti-E-Cadherin (Abcam, ab231303), anti-N-Cadherin (CST, 4061S), anti-Vimentin (Abcam, ab137321), anti-Snail (CST, 3879), anti-AKT (CST, 2938S), anti-phospho-AKT (Ser473) (CST, X4060S), anti-mTOR (CST, 2972), anti-phospho-mTOR (Ser2448) (CST, 2971), anti-β-catenin (CST, 9562), anti-phospho-β-catenin (Ser675) (CST, 9567S).

### CCK8 (cell counting kit-8) and colony formation assay

Transduced cells were plated in 96-well plates at a density of 1 × 10^3^ cells/well and cell viability was investigated after 24, 48, 72, 96, and 120 h. 10 μl of CCK-8 solutions (Dojindo, Japan) was added to each well and then put the 96-well plate into cell incubator. After 40 min, the absorbance at 450 nm was recorded.

For colony formation assays, a total of 1 × 10^3^ cells was seeded into each well of the 6-well plate and cultured for 14 days. Medium was replaced each 3 days. Cells were washed with PBS, fixed with 4% paraformaldehyde, and stained with crystal violet (Beyotime, China). After staining, photographs were taken, and numbers of colonies were counted.

### Wound healing assay

Wound healing assays were performed as previously reported. In brief, cells were seeded into 6-well plates to obtain a complete monolayer. Then 200 μl pipette tip was used to make wounds across the cell monolayer. After removing dead cells with PBS, cells were cultured with serum-free DMEM medium. Each wound was photographed at the indicated time and wound healing rate was calculated using Image J software.

### Cell invasion assays

Invasion assay was performed in transwell chambers coated with Matrigel (BD Biosciences, USA). Cells were seeded onto the upper chambers at a density of 1 × 10^4^/well; the lower chamber was filled with 500 μl DMEM medium containing 10% FBS. After 24 h, the cells on the bottom side of the filter were fixed in 4% paraformaldehyde, stained with 0.1% crystal violet, and 10 microscopic fields were quantified under a light microscope. Results were obtained from three independent experiments.

### In vivo experiments

A total of 24 male BALB/c nude mice (6–8 weeks, 20–22 g) were used in this study. To establish subcutaneous xenograft tumor model, 1 × 10^6^ cells were injected on sides of the flank of mice (*n* = 6 per group). Tumor size were observed each 5 days. 28 days after injection, the mice were sacrificed with tumor size and tumor weight being measured. To establish pulmonary metastasis model, 2 × 10^6^ HCC cells were inoculated via caudal vein (*n* = 6 per group). After 28 days, the mice were sacrificed and the metastatic rate of lung in mice were counted. All the protocols of animal experiments have been approved by the Animal Care Committee of Fudan University (Shanghai, China).

#### Co-immunoprecipitation assay and mass spectrometry assay

For co-immunoprecipitation assay, HCC cells lysate was centrifuged for 15 min at 10,000 g, and the supernatant was incubated with Protein A/G plus-agarose beads for 1 h at 4 °C. After centrifugated for 15 min at 14000 g, primary antibody was added to the supernatant and incubated overnight. Then, Protein A/G plus-agarose beads (Pierce Biotechnology, Rockford, IL, USA) were added to the mixture for 2 h. The precipitates were boiled and loaded onto 10% SDS-PAGE gels and the potential interacting proteins were detected by using mass spectrometry assay (MS). The mass spectrometry results were identified using SEQUEST (v.28, Thermo Electron) against the Human International Protein Index database.

### Luciferase reporter gene assay

The TRIP13 3′UTR sequence and the target-site mutant 3′UTR sequence were amplified and cloned into the pmirGLO DUAL-luciferase reporter (Promega). HCC cells were co-transfected with TRIP13 3′-UTR-wt or TRIP13 3′-UTR-mut, and miR-192-5p mimics, miR-control using the Lipofectamine 2000 reagent (Invitrogen, USA). Following cultivation for 48 h, the transfected cells were collected and detected for luciferase activity using a dual luciferase reporter assay kit (Promega, USA) based on the manufacturer’s instructions.

### Statistical analysis

Student’s t-test was used for two groups comparisons and one-way anova test was used for multiple comparisons. Log-rank test and cox regression model were used to analyze patient survival. All statistical analyses were performed using SPSS 20.0 software (IBM, USA). Data are shown as mean ± SEM and *P* < 0.05 was considered significant.

## Results

### Identification of TRIP13 as a potential target in HCC

**Fig. 1:**
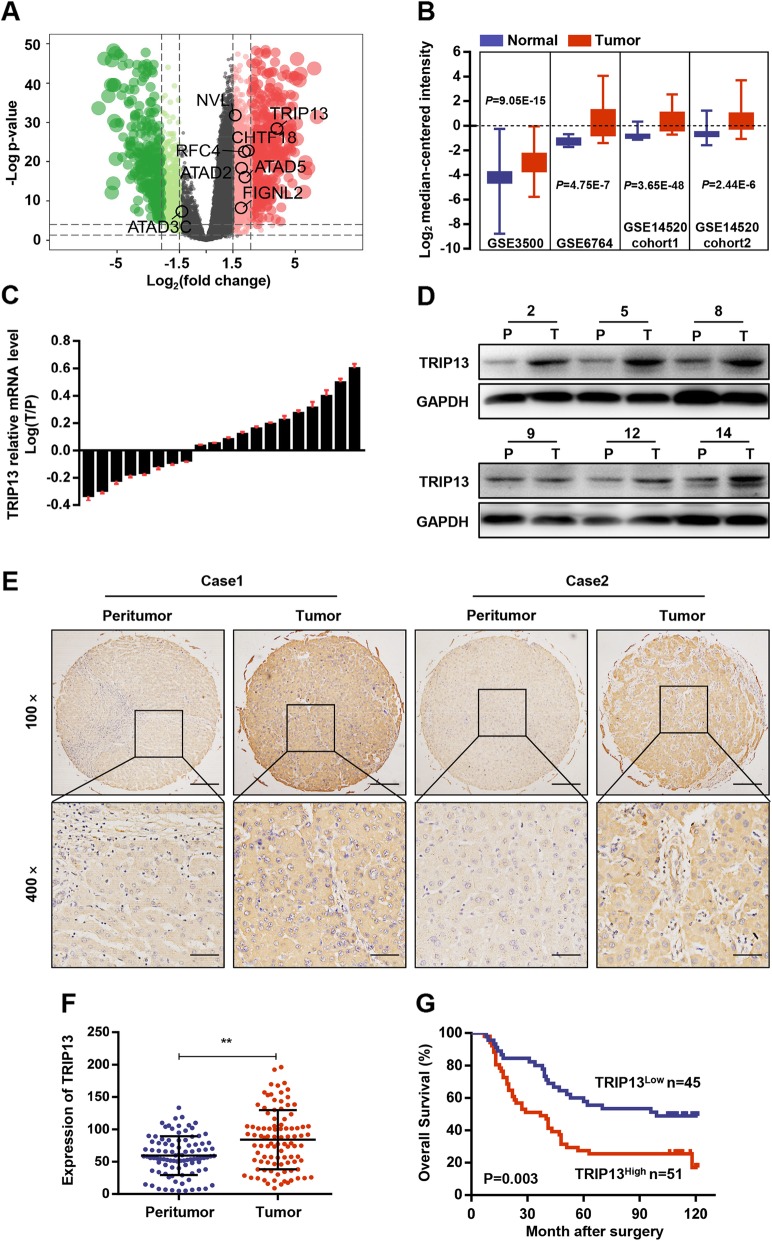
Upregulation of TRIP13 in HCC. **a** Volcano plot indicated differentially expressed AAA ATPase genes in TCGA LIHC dataset. **b** Studies from GEO datasets showed higher mRNA expression of TRIP13 in HCC tissues. **c**-**d** The mRNA and protein expression of TRIP13 in 15 pairs of HCC (T) and peritumor tissues (P) were analyzed by real-time PCR and western blot. The representative images were shown. **e** The expression of TRIP13 was detected by IHC in a TMA including 96 HCC cases and typical photos were presented. **f** IHC results presented the increase of TRIP13 protein in HCC compared with peritumor samples. G Survival analysis of TRIP13 expression in 96 HCC patients. **P < 0.01

To screen AAA ATPase family members involved in HCC development, RNA sequencing and clinical data from the TCGA-LIHC dataset, including 369 HCC samples and 50 normal samples, were firstly analyzed. Differential gene expression analysis revealed 3155 DEGs (differentially expressed genes), among which six AAA ATPase genes (TRIP13, CHTF18, RFC4, ATAD2, FIGNL2, ATAD5) were significantly overexpressed in tumor samples compared to levels in normal samples (Fig. 1a). Then, overall survival analysis was performed based on each differentially-expressed AAA ATPase gene and the results showed that TRIP13, the gene with the most significant expression differences, had the highest prognostic significance (Additional file 1: Figure S1). To validate these results, we further identified TRIP13 mRNA expression in the Oncomine database. Consistently, TRIP13 was observed elevated in four HCC datasets (GSE3500, *P* = 9.05E-15; GSE6764, *P* = 4.75E-7; GSE14520 Cohort 1, *P* = 3.65E-48; GSE14520 Cohort 2, *P* = 2.44E-6) (Fig. 1b). Therefore, we speculated that TRIP13 might play certain roles in the HCC and explored its potential functions in the following studies.

### TRIP13 is highly expressed in HCC and correlated with poor prognosis

Next, TRIP13 expression and its clinical significance were assessed in our own cohorts of HCC patients. We examined TRIP13 expression in 20 cases of fresh frozen HCC and matched peritumor tissues. Results revealed that TRIP13 was elevated in HCC tissues at both the mRNA and protein levels (Fig. 1c-d and Additional file 3: Figure S3A). Subsequently, immunohistochemical analysis was performed on a TMA comprising 96 HCC and matched adjacent samples. The results demonstrated that TRIP13 expression was higher in HCC than that in peritumor tissue (Fig. 1e and f).

To investigate the clinical significance of TRIP13, 96 HCC patients were divided into two groups according to the TRIP13 expression, as described in the Materials and Methods section. We assessed the correlation between the TRIP13 expression and clinicopathological parameters, and found that high TRIP13 levels were positively associated with advanced TNM stages (*p* = 0.029), larger tumor size (*p* = 0.014), and lower differentiation (*p* = 0.031). Furthermore, Kaplan–Meier survival analysis indicated that patients with high TRIP13 expression had significantly lower overall survival rates compared to those without (Fig. 1g). Finally, Cox multivariate regression analysis showed that TRIP13 was an independent prognostic factor for HCC patients (Table 1).

**Table 1:** Univariate and multivariate analysis of TRIP13 in OS of 96 HCC patients

Variables		Univariate analysis			Multivariate analysis		
		HR	95%CI	*P*-value	HR	95%CI	*P*-value
Age (year)	<50 vs. ≥50	0.854	0.519-1.408	0.537	NA		
Gender	male vs. female	2.159	1.026-4.540	0.042	NA		
Tumor size (cm)	≤5 *vs*. >5	1.806	1.087-3.001	0.004^a^	1.589	0.951-2.655	0.077
Tumor number	mutiple vs. single	0.693	0.381-1.258	0.227	NA		
Tumor differentiation	I-II *vs*. III-IV	1.191	0.674-2.104	0.548	NA		
Microvascular invasion	yes *vs*. no	2.589	1.562-4.290	0.000224^a^	2.345	1.410-3.900	0.001^a^
HBsAg	positive *vs*. negative	0.699	0.355-1.377	0.301	NA		
History of cirrhosis	yes *vs*. no	1.716	0.843-3.494	0.137	NA		
TNM stage	I-II *vs*. III-IV	1.555	0.927-2.608	0.095	NA		
TRIP13	low *vs*. high	2.125	1.265-3.571	0.004^a^	1.816	1.074-3.070	0.026^a^

Note: Univatiate and multivariate analysis, Cox proportional hazards regression model. *OS* Overall survival time, *HR* Hazard ratio, *CI* Confidence interval, *NA* Not applicable

^a^Statistically significant

### TRIP13 promotes HCC cell proliferation, migration, and invasion in vitro

**Fig. 2:**
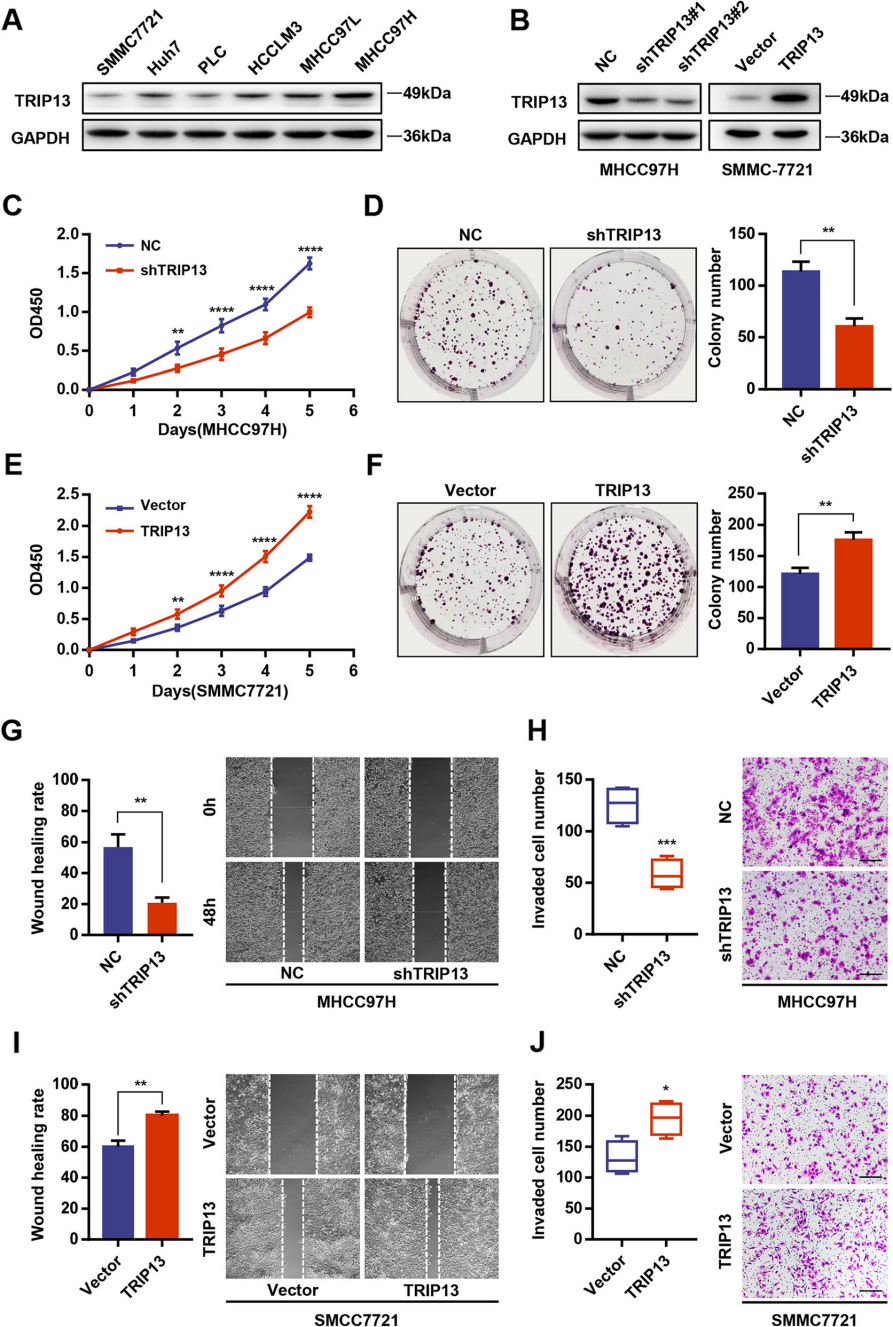
TRIP13 promoted cell proliferation, migration and invasion of HCC cells in vitro. **a** Western blot analysis of TRIP13 in six different HCC cell lines. **b** The efficiency of transfection in SMMC7721 and MHCC97H cell lines were confirmed by western blot. **c**-**d** Knockdown of TRIP13 in MHCC97H cells inhibited cell proliferation as determined by CCK-8 (**c**) and colony formation assays (**d**). **e**-**f** Overexpression of TRIP13 in SMMC7721 cells promotes cell proliferation as determined by CCK-8 (**e**) and colony formation assays (**f**). **g**-**h** Interference with TRIP13 expression in MHCC97H cells inhibited cell migration and invasion as determined by wound healing assay (**g**) and transwell assay (**h**). **i**-**j** Overexpression of TRIP13 in SMMC7721 cells promotes cell migration and invasion as determined by wound healing assay (**i**) and transwell assay (**j**). *P < 0.05, **P < 0.01, ***P < 0.001, ****P < 0.0001

To evaluate the biological functions of TRIP13, TRIP13-shRNAs were used to knock down expression in MHCC97H cells, which had the highest endogenous TRIP13 expression, whereas TRIP13 was overexpressed through transfected with pLV5-TRIP13 lentivirus in SMMC7721 cells, with the lowest endogenous levels (Fig. 2a and b). By performing CCK-8 and colony formation assays, we found that TRIP13 silencing remarkably attenuated MHCC97H cell viability and colony formation (Fig. 2c and d). In contrast, TRIP13 overexpression promoted cell viability and colony formation of SMMC7721 cells, supporting the contention that TRIP13 facilitates HCC cell proliferation (Fig. 2e and f). Meanwhile, the migratory and invasive abilities of MHCC97H TRIP13-knockdown cells was dramatically decreased based on wound healing and transwell assays (Fig. 2g and h); accordingly, TRIP13-overexpressing SMMC7721 cells exhibited enhanced migratory and invasive capacity (Fig. 2i and j). Thus, elevated TRIP13 promotes tumor cell proliferation, migration, and invasion in HCC cells.

### TRIP13 promotes tumor growth and pulmonary metastasis in vivo

**Fig. 3:**
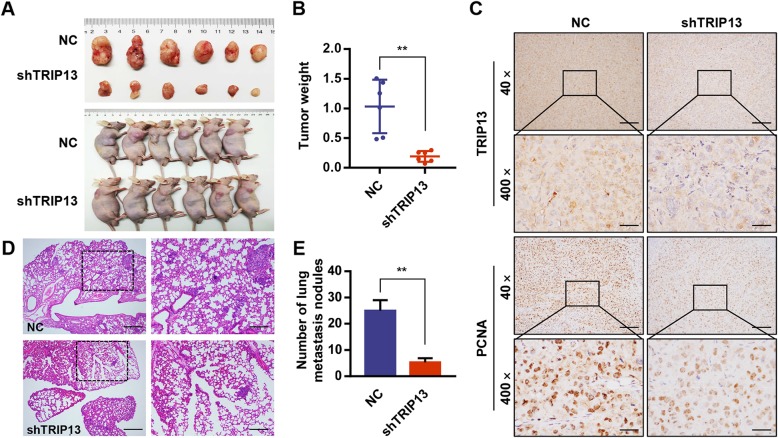
TRIP 13 silencing inhibited tumor growth and lung metastasis in vivo. **a** MHCC97H cells with different TRIP13 expression were subcutaneously transplanted the xenograft into BALB/c nude mice (*n* = 6/group) and tumor sizes were measured after 4 weeks. **b** Tumor weights were measured. **c** Tumors were formalin-fixed, paraffin-embedded, sliced and stained with TRIP13 and PCNA. Representative images of each group are presented. **d**-**e** MHCC97H cells with different TRIP13 expression were injected into BALB/c nude mice (*n* = 6/group) through the tail vein to establish lung metastasis models. The number of lung metastatic nodules in each group was counted under the microscope. Representative H&E staining images of lungs were shown. **s < 0.01

To investigated the effects of TRIP13 in tumor growth, MHCC97H cells with stable TRIP13 knockdown or negative control cells were injected subcutaneously into the right flank of immunodeficient nude mice. Tumor volumes were measured each five days. Two weeks later, the mice were sacrificed to determine the tumor weights and volumes. The data indicated that silencing TRIP13 greatly suppressed tumor growth, as shown by reduced tumor sizes and weights in the MHCC97H-shTRIP13 group compared to those in the negative control group (Fig. 3a and b). Additionally, immunohistochemistry for TRIP13 and PCNA (a widely accepted marker of cell proliferation) showed that TRIP13 silencing led to a substantial reduction in PCNA protein levels, corroborating the growth-promoting effects of TRIP13 in HCC (Fig. 3c).

Furthermore, to determine the effect of TRIP13 on tumor metastasis, we performed histological examination of formalin-fixed, paraffin-embedded lung tissue sections from mice injected with MHCC97H-shRNA cells via the tail vein. Results indicated that sections from the MHCC97H-shRNA group had fewer lung metastatic nodules than those from the negative control group (Fig. 3d and e). Consequently, these findings demonstrate that silencing TRIP13 inhibits HCC lung metastasis in vivo.

### TRIP13 regulates migration and invasion via EMT in HCC cells

**Fig. 4:**
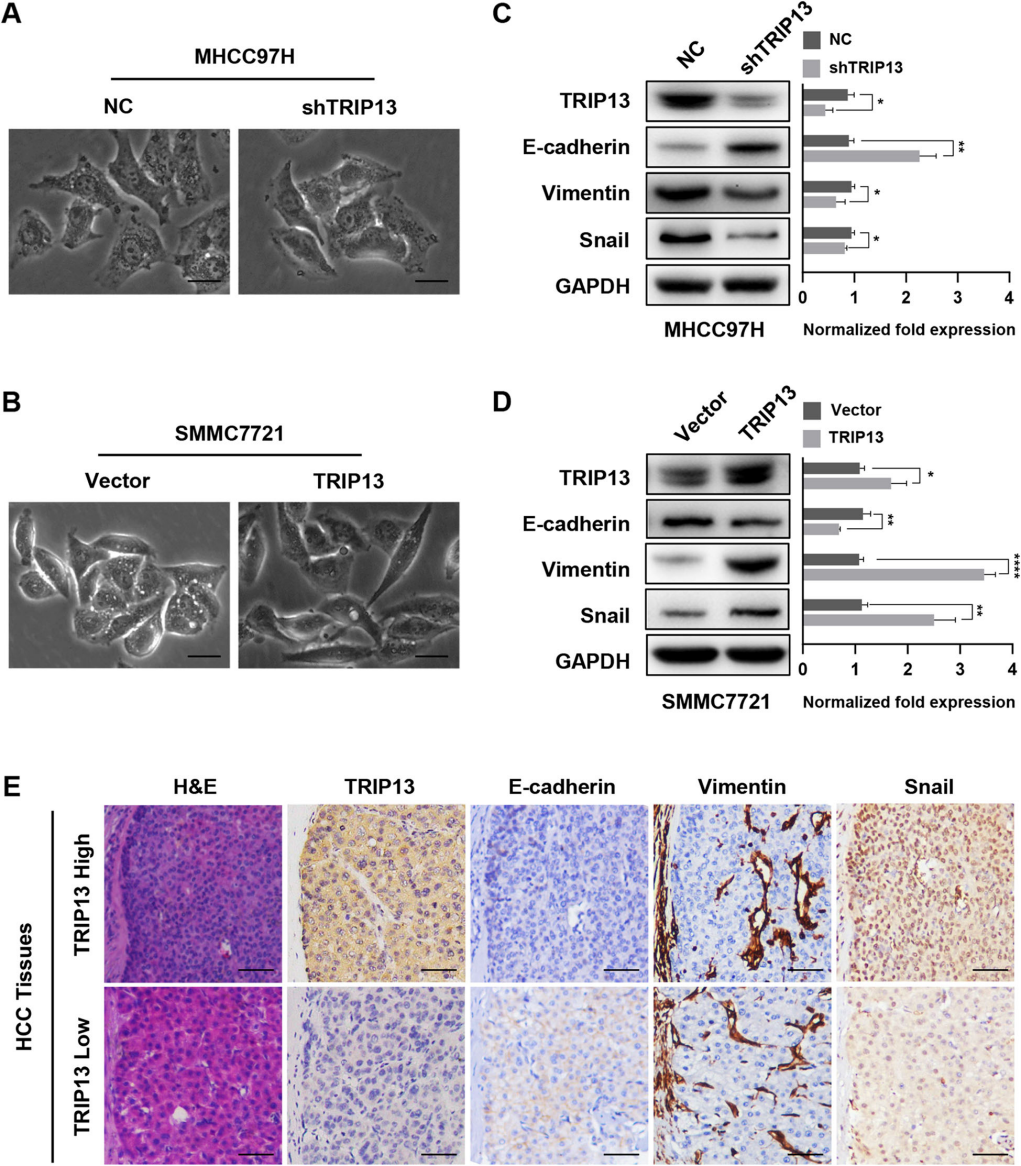
TRIP13 induced EMT in HCC cells. **a**-**b** The morphology of HCC cells with different TRIP13 expression. **c**-**d** Western blot analysis of the EMT markers (E-cadherin, N-cadherin, vimentin) in MHCC97H or SMMC7721 cells with knockdown or overexpressing TRIP13. **e** IHC staining of EMT markers (E-cadherin, Vimentin, Snail) in serial section of human HCC tissues. *P < 0.05, **P < 0.01, ***P < 0.001, ****P < 0.0001

As EMT is recognized as an important event associated with carcinoma progression, we speculated that TRIP13 might participate in this process. We first observed the morphology of HCC cells with different levels of TRIP13 expression. As shown in Fig. 4a and Fig. 4b, SMMC7721-vector and MHCC97H-shTRIP13 cells exhibited a cobble stone-like appearance, typical of normal epithelial cells, whereas SMMC7721-TRIP13 and MHCC97H-shNC cells adopted a spindle-like, fibroblastic morphology. Next, we measured changes in several EMT markers in the aforementioned transfected HCC cells. Western blot analysis revealed that TRIP13 knockdown could enhance the level of the epithelial marker E-cadherin and reduce levels of the mesenchymal marker vimentin and snail in MHCC97H cells (Fig. 4c); conversely, overexpression of this gene yielded the opposite effect on these EMT markers (Fig. 4d). Furthermore, IHC analysis of serial sections showed that HCC cells with higher levels of TRIP13 over-expressed vimentin and snail and lost E-cadherin expression, strongly suggesting that cells with elevated levels of this marker had underwent EMT (Fig. 4e). Thus, we conclude that TRIP13 promotes the metastasis of HCC via inducing EMT in HCC cells.

### TRIP13 induces EMT through the AKT/mTOR pathway

**Fig. 5:**
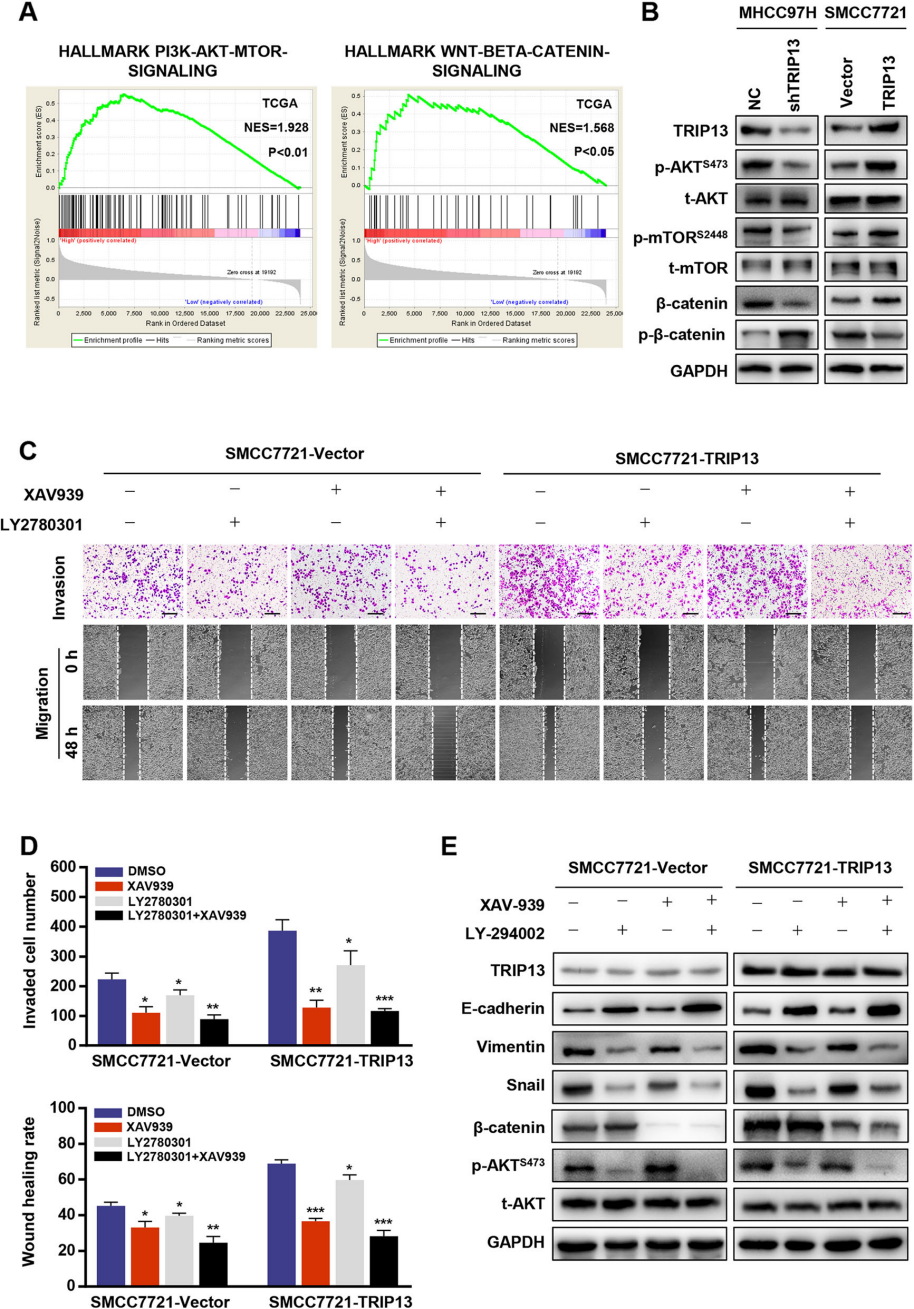
TRIP13 altered activation of PI3K/AKT/mTOR pathway to induce cell EMT. **a** Gene set enrichment analysis of TCGA LIHC dataset showed enrichment of PI3K/AKT/mTOR pathway and Wnt/β-catenin pathway gene panels in the TRIP13 high expression group. **b** Reprehensive genes of PI3K/AKT/mTOR pathway and Wnt/β-catenin pathway were detected in TRIP13 knockdown or overexpression cells. **c**-**d** Cells were treated with XAV939 or LY294002 and cell migration and invasion were measured. **e** Cells were treated with XAV939 or LY294002 and the levels of EMT markers were analyzed. *P < 0.05, **P < 0.01, ***P < 0.001

To decipher the mechanism underlying TRIP13-induced HCC metastasis, Gene Set Enrichment Analysis (GSEA) based on the TCGA-LIHC dataset was performed. The results indicated that TRIP13 expression was positively related with genes up-regulated by activation of the PI3K/AKT/mTOR pathway or Wnt/β-catenin pathway, indicating that TRIP13 may participate in the activation of these signaling pathways (Fig. 5a). As such, we investigated some typical markers of the two pathways in transfected HCC cells. As shown in Fig. 5b, western blot analysis showed that silencing TRIP13 repressed the expression levels of p-AKT^S473^, p-mTOR^S2884^, and β-catenin. In contrast, up-regulating TRIP13 increased these markers in HCC cells. These results confirmed that TRIP13 activates both PI3K/AKT/mTOR and Wnt/β-catenin signaling in HCC cells.

We further determined which signaling mechanism plays a critical role in TRIP13-induced HCC metastasis using the AKT inhibitor LY294002 and Wnt inhibitor XAV939. Notably, LY294002 abolished the stimulatory effect of TRIP13 over-expression on migration and invasion in HCC cells and caused corresponding changes in E-cadherin, vimentin, and snail expression, whereas XAV939 had no significant influence on cellular or EMT markers (Fig. 5c–e). These data indicate that TRIP13 promotes HCC metastasis through the activation of AKT/mTOR signaling.

### TRIP13 interacts with ACTN4 to activate the AKT/mTOR pathway

**Fig. 6:**
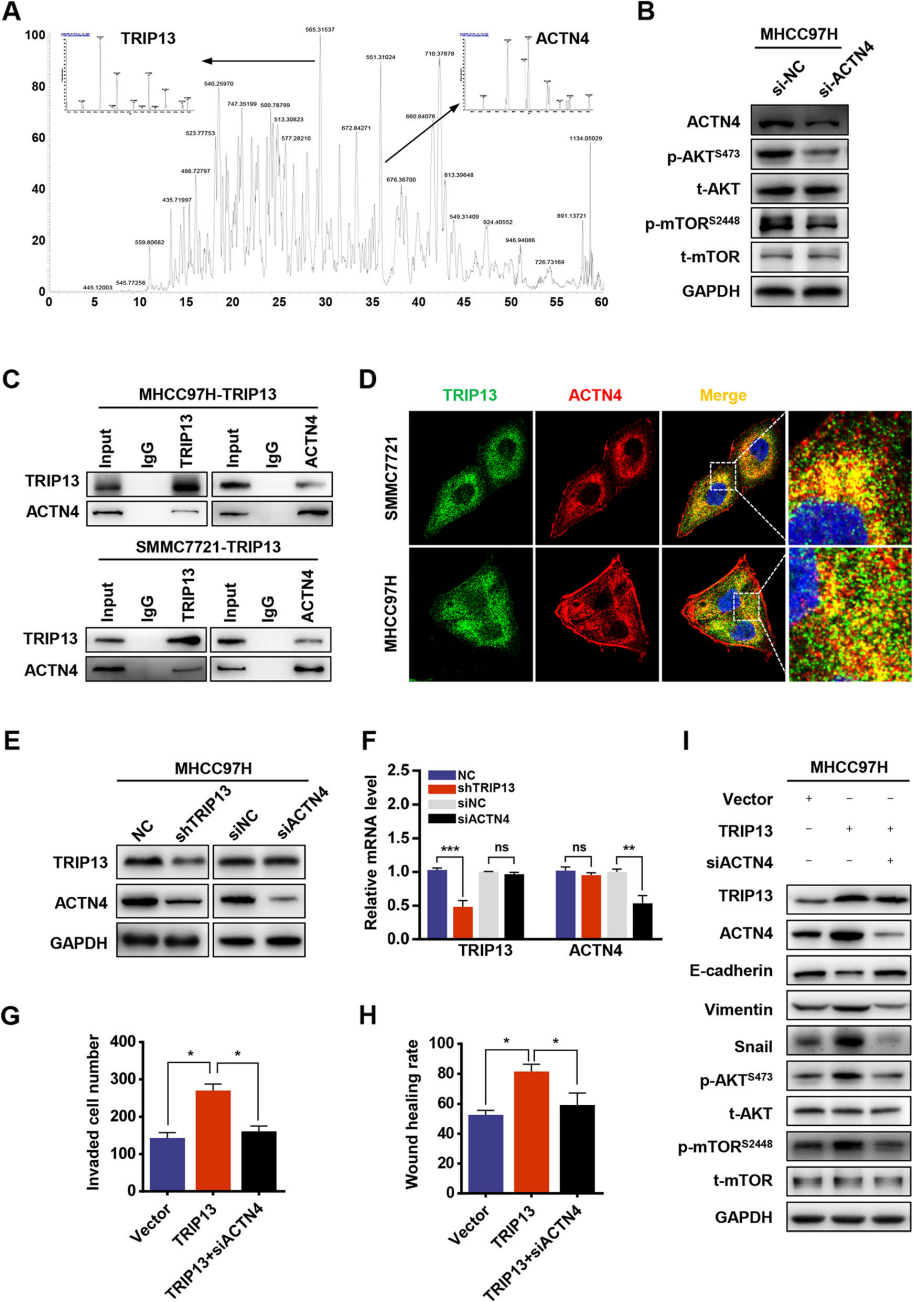
TRIP13 activates AKT/mTOR pathway by interacting with ACTN4. **a** ACTN4 was identified as a binding partner of TRIP13 by combining Co-IP and MS. **b** The AKT/mTOR pathway was affected by ACTN4 interference. **c** Co-IP was used to validate the interaction of TRIP13 and ACTN4. **d** Immunofluorescence used to verify the colocalization of TRIP13 and ACTN4. **e**-**f** qRT-PCR and western blot analysis of TRIP13 or ACTN4 mRNA expression in HCC cells transfected with shTRIP13 or siACTN4. **g**-**h** Cell migration and invasion were measured in the indicated cells. **i** EMT markers and reprehensive genes of AKT/mTOR pathway were measured in the indicated cells by western blot. *P < 0.05, **P < 0.01, ***P < 0.001

To further explore the molecular mechanism through which TRIP13 activates the AKT/mTOR pathway, mass spectrometry (MS) was performed to find potential TRIP13-binding proteins in two HCC cell lines. As a result, 74 and 59 proteins were identified in SMCC7721 and MHCC97H cells respectively, and eight proteins overlapped (Additional file 2: Figure S2A). Among candidate TRIP13-interacting partners, we selected ACTN4 for further investigation as prior reports showed that it is involved in cancer cell EMT and AKT signaling [12, 13] (Fig. 6a). By transfecting MHCC97H cells with siACTN4 and examining AKT/mTOR-associated gene signatures, ACTN4 was confirmed to activate this signaling pathway in HCC cells (Fig. 6b). The interaction between TRIP13 and ACTN4 was further verified by co-immunoprecipitation and confocal assays in both TRIP13-overexpressing SMMC7721 and MHCC97H cells (Fig. 6c and d). Moreover, we found that TRIP13 expression was positively correlated with ACTN4 in HCC tissues and TRIP13 knockdown significantly downregulated ACTN4 protein, whereas the knockdown of ACTN4 had no effect on the expression of TRIP13 (Fig. 6e and Fig. S3B-D). In addition, TRIP13 silencing did not influence ACTN4 mRNA levels (Fig. 6f). This suggests that ACTN4 is downstream of TRIP13 and that its expression is regulated by TRIP13 at the protein level. Furthermore, we observed that ACTN4 knockdown in MHCC97H cells abrogated cell invasion, migration, and EMT induced by TRIP13 over-expression (Fig. 6g–i). Collectively, these data demonstrate that TRIP13 interacts with ACTN4 to activate the AKT/mTOR pathway and confer a mesenchymal phenotype.

### miR-192-5p targets TRIP13 and inhibits TRIP13 expression

**Fig. 7:**
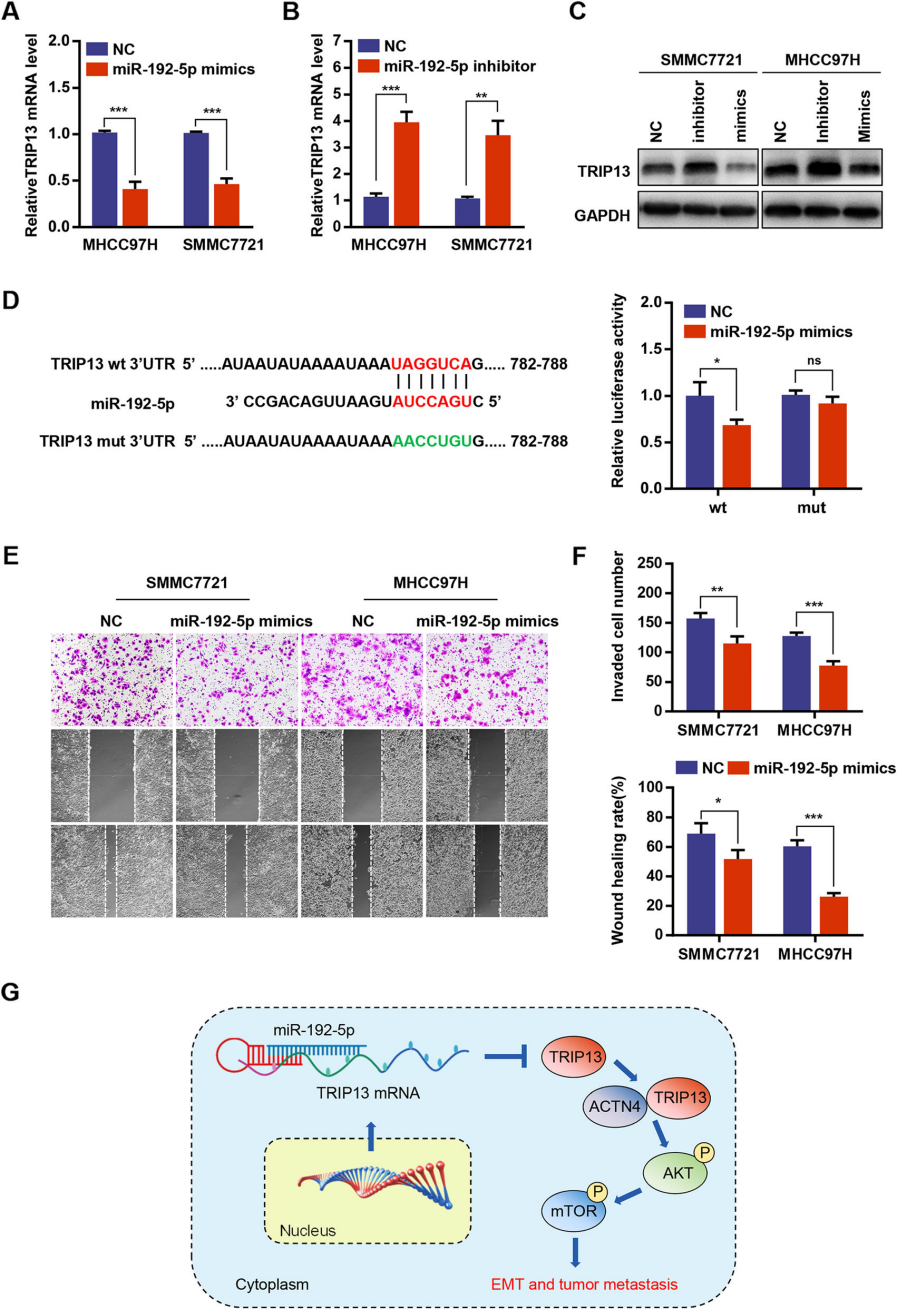
miR-192-5p inhibited TRIP13 expression in HCC cells. **a**-**c** qRT-PCR and western blot analysis of TRIP13 mRNA in HCC cells transfected with miR-192-5p mimics or miR-192-5p inhibitor. **d** The putative miR-192-5p binding sequence in the 3′-UTR of TRIP13 (left penal). Luciferase activity of 293 T cells co-transfected with WT or MUT luciferase reporter plasmids and miR-192-5p mimics or negative control (right penal). **e**-**f** Cell migration and invasion were measured in HCC cells transfected with miR-192-5p mimics or negative control. **g** A model depicting the major molecular mechanisms of the miR-192-5p–TRIP13–ACTN4–AKT/mTOR axis in HCC. *P < 0.05, **P < 0.01, ***P < 0.001

The mechanism responsible for TRIP13 upregulation in HCC was then explored. Using the publicly available algorithms miRanda, microT, miRmap and PITA, four microRNAs were predicted as potential upstream regulators of TRIP13 (Additional file 2: Figure S2B). Since miR-192-5p level is negatively correlated with TRIP13 expression in TCGA LIHC datasets and has been proved to be involved in the progression of HCC, it was chosen for further studies [14] (Additional file 3: Figure S3E). qRT-PCR and western blot analysis revealed that miR-192-5p mimics could reduce expression levels of TRIP13, whereas the suppression of miR-192-5p by its specific inhibitor led to increased TRIP13 (Fig. 7a–c). Moreover, a putative binding site for miR-192-5p in the TRIP13 3′UTR (untranslated region) was predicted based on the TargetScan database. Dual luciferase reporter assays showed that the overexpression of miR-192-5p markedly inhibited reporter activity from the TRIP13 3′UTR but not from the mutant 3′UTR (Fig. 7d). Furthermore, invasion and migration assays were performed, and the results indicated that miR-192-5p up-regulation decreased, whereas miR-192-5p silencing increased, the invasion and migration of HCC cells (Fig. 7e). These results indicate that miR-192-5p inhibits the expression of TRIP13, as well as invasion and migration, in HCC cells.

## Discussion

In recent years, proteins of AAA ATPase family members received increasing attention for their critical roles in tumor formation and progression. Through reanalyzing gene expression profiles from the TCGA and GEO databases, we found that TRIP13 was the most prominent differentially expressed AAA ATPase gene in HCC and was an independent prognostic factor for HCC patients. Our data showed that TRIP13 facilitated the invasion and metastasis of HCC through interacting with ACTN4 to drive the activation of AKT/mTOR pathway.

TRIP13 was originally recognized as a participant in meiotic recombination, spindle assembly checkpoint, and DNA repair [15–17]. Actually, as a AAA ATPase, which can facilitate the assembly or degradation of protein complexes, TRIP13 has been reported to be significantly up-regulated in multiple types of tumors and involved in tumorigenesis by binding various proteins [18, 19]. Bannerjee and his colleagues first verified that TRIP13 is an oncogene and demonstrated that it induces chemoresistance in head and neck cancer by interacting with the DNA-PK complex to enhance non-homologous end joining [17]. Ma and Tao et al. showed that TRIP13 binds with the MAD2 (a cell cycle checkpoint protein) to induce the proteasomal degradation of this protein or convert it to its open conformation, thereby promoting drug resistance and tumor cell proliferation [16, 20]. Aside from its important role in tumor cell proliferation, TRIP13 was also reported to promote tumor cell migration and invasion [21, 22]. Sheng et al. showed that TRIP13 interacts with YWHAZ, which mediates G2-M transition and EMT, and thus promoting CRC cell proliferation, invasion and migration [23]. However, based on high throughput analysis, we are the first to find that TRIP13 interacts with ACTN4 and induce EMT in an ACTN4-dependent manner. This is a new mechanism for TRIP13 to promote tumor metastasis.

ACTN4, known as an actinin-binding protein, is important for the regulation of cytoskeletal integrity and cell movement. Previous studies showed that it has a critical role in the pathogenesis of cancer due to its ability to promote cancer cell invasion and metastasis, as well as signaling pathways regulation [13, 24–26]. An et al. indicated that ACTN4 induces EMT through the AKT signaling pathway in cervical cancer [12]. Moreover, ACTN4 is an AKT1-binding partner that up-regulates AKT phosphorylation [27]. Consistently, our experiments showed that ACTN4 activates AKT/mTOR signaling in HCC cells. Furthermore, ACTN4 knockdown partly abolished TRIP13-induced EMT and activation of the AKT/mTOR pathway. Taking together, these results indicate that TRIP13 triggers AKT/mTOR signaling to promote EMT by interacting with ACTN4 and regulating its expression. In future research, we will focus on the mechanism through which TRIP13 up-regulates ACTN4 expression in HCC cells. Additionally, different with our result, an earlier research reported that TRIP13 facilitates HCC cell growth and metastasis through activating of TGF-β1/smad3 signaling [28]. Considering AKT is another target of TGF-β1, we conjectured that there may exist a network of the two signaling pathway genes that promote HCC progression synergistically.

MicroRNAs (miRNAs) are small non-coding RNAs of approximately 20 nucleotides in length. By binding to mRNAs at the 3′UTR, they inhibit mRNA translation or lead to mRNA degradation [29, 30]. During the past few years, miRNAs have been found to be aberrantly expressed in tumor tissues and to contribute to the occurrence of cancer. More importantly, miRNAs have been considered regulators of oncogenes or tumor suppressor genes [30, 31].

In the present study, miR-192-5p was verified to be the upstream regulator of TRIP13 and was found to suppress cell motility in HCC cells. Consistent with our results, previous studies have reported low miR-192-5p levels in cancer, and that it suppresses the growth and metastasis of osteosarcoma, HCC, colon cancer and bladder cancer [14, 32–35].

## Conclusion

Our findings identify a TRIP13/ACTN4/AKT/mTOR axis in HCC cells, and show that this pathway drives HCC progression. In addition, we show that TRIP13 is regulated by miR-192-5p, which might be responsible for the high expression of TRIP13 in HCC. Our study could help to better understand the mechanisms underlying HCC progression; moreover, as a prognostic predictor, TRIP13 might be a potential target for HCC therapy.

## Additional files

Additional file 1: **Figure S1.** Survival analysis of six differentially expressed AAA ATPase genes. Patients were classified into two groups according to gene expressions. Kaplan–Meier survival curves and log-rank test for different groups were performed. (TIF 9035 kb)

Additional file 2: **Figure S2.** Prediction of TRIP13 binding proteins and microRNAs. **A** Venn chart showed the number of binding partners of TRIP13 between SMMC7721 and MHCC97H, and 8 overlapped proteins were included in the diagram. Eight overlapped proteins are listed in the Table. **B** Venn chart showed the number of binding microRNAs of TRIP13 predicted by different databases. Four overlapped microRNAs are listed in the Table. (TIF 9913 kb)

Additional file 3: **Figure S3. A** Statistical analysis of TRIP13 expression in HCC and peritumor samples. **B** Representative images of ACTN4 IHC in xenograft mouse tumors samples. **C**-**D** TRIP13 expression is positively correlated with ACTN4 expression in HCC tissue microarray. **E** Patients were classified into two groups according to gene expressions. Kaplan–Meier survival curves and log-rank test for different groups were performed. (TIF 20535 kb)

## Data Availability

The data supporting our conclusion were obtained from the TCGA database (https://cancergenome.nih.gov) and Oncomine database (https://www.oncomine.org).

## Abbreviations

AAA ATPase: ATPase associated with a variety of cellular activities
CCK8: Cell counting kit-8
DEGs: differently express genes
EMT: Epithelial–mesenchymal transition
GEO: Gene expression omnibus
GSEA: Gene set enrichment analysis
HCC: Hepatocellular carcinoma
miRNA: microRNA
MS: Mass spectrometry
PBS: Phosphate buffer saline
qRT-PCR: Quantitative real-time polymerase chain reaction
TCGA-LIHC: The Cancer Genome Atlas Liver Hepatocellular Carcinoma
TRIP13: thyroid hormone receptor interactor 13
UTR: Untranslated region
