# Correction to: Elevated TRIP13 drives the AKT/mTOR pathway to induce the progression of hepatocellular carcinoma via interacting with ACTN4

**DOI:** 10.1186/s13046-019-1454-y

**Published:** 2019-10-31

**Authors:** Meng-Xuan Zhu, Chuan-Yuan Wei, Peng-Fei Zhang, Dong-Mei Gao, Jie Chen, Yan Zhao, Shuang-Shuang Dong, Bin-Bin Liu

**Affiliations:** 1 Liver Cancer Institute, Zhongshan Hospital, Fudan University and Key Laboratory of Carcinogenesis and Cancer Invasion, Ministry of Education, 180 Fenglin Road, Shanghai, 200032 China; 2 Department of Liver Surgery, Liver Cancer Institute, Zhongshan Hospital, Fudan University, 180 FengLin Road, Shanghai, 200032 China

**Correction to: J Exp Clin Cancer Res (2019) 38: 409**

**https://doi.org/10.1186/s13046-019-1401-y**

In the original publication of this article [1], the author would like to revise Fig. 4.

In this article, the authors explored the relationship between the expression level of TRIP13 and EMT markers (E-cadherin, vimentin, snail) in their HCC tissue microarray (Fig. 4e), and this tissue microarray was also used in their preciously published article in Journal of Experimental & Clinical Cancer Research (Overexpression of RNF38 facilitates TGF-β signaling by Ubiquitinating and degrading AHNAK in hepatocellular carcinoma. Doi: 10.1186/s13046-019-1113-3. (Fig. 3e)). Unfortunately, the authors of these two articles selected and displayed immunohistochemical images of the same patient without knowing it. In order to make the article more rigorous, the authors of this article replaced Fig. 4 with another patient’s immunohistochemical images. The revised Fig. 4 is shown below:

**Figure 1:**
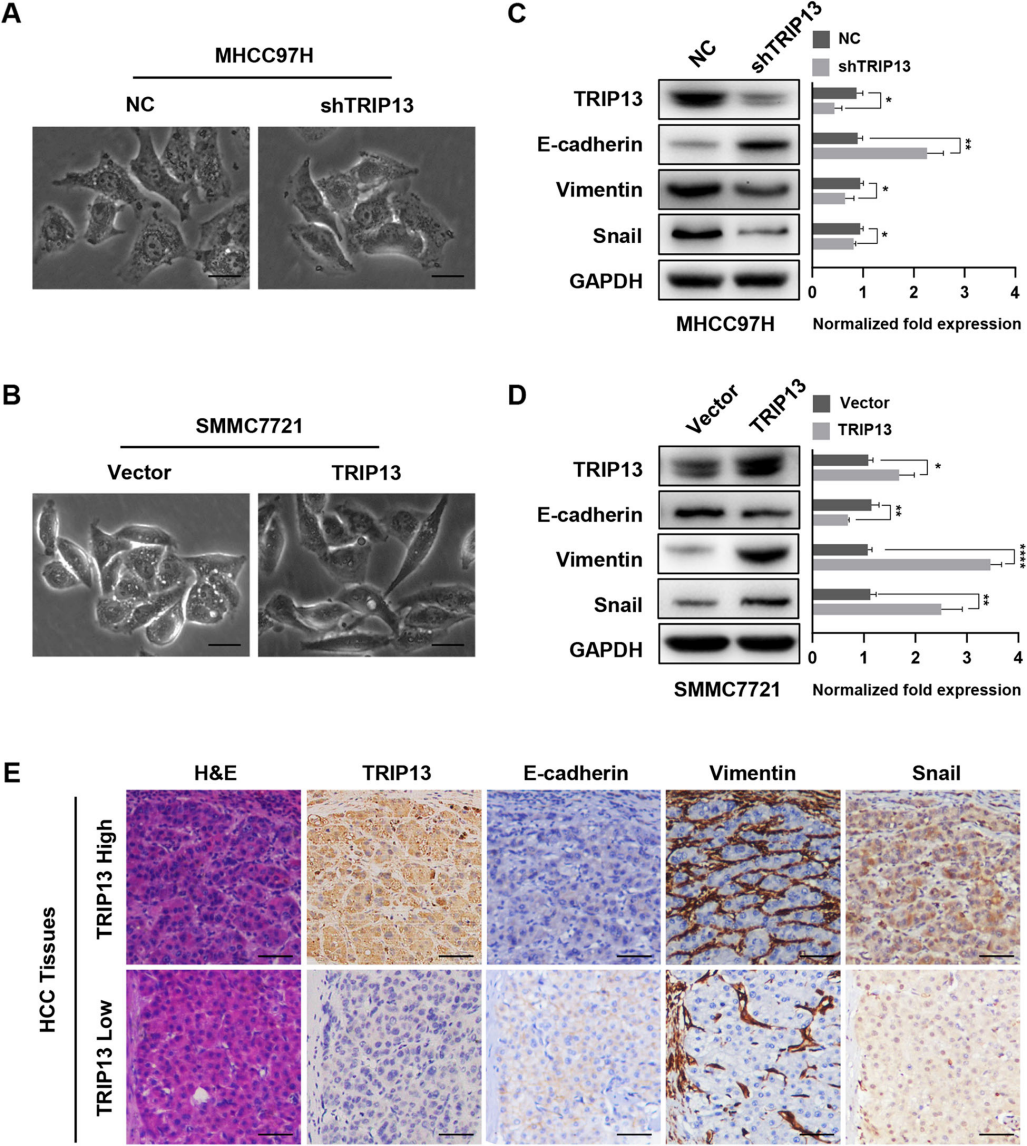
Fig 4

The authors sincerely apologize for the inconvenience caused to the readers.
