# Supplementary figures and images for: Elevated TRIP13 drives the AKT/mTOR pathway to induce the progression of hepatocellular carcinoma via interacting with ACTN4
Source: J Exp Clin Cancer Res. 2019 Sep 18;38:409. doi: 10.1186/s13046-019-1401-y (PMC6749659; doi:10.1186/s13046-019-1401-y)

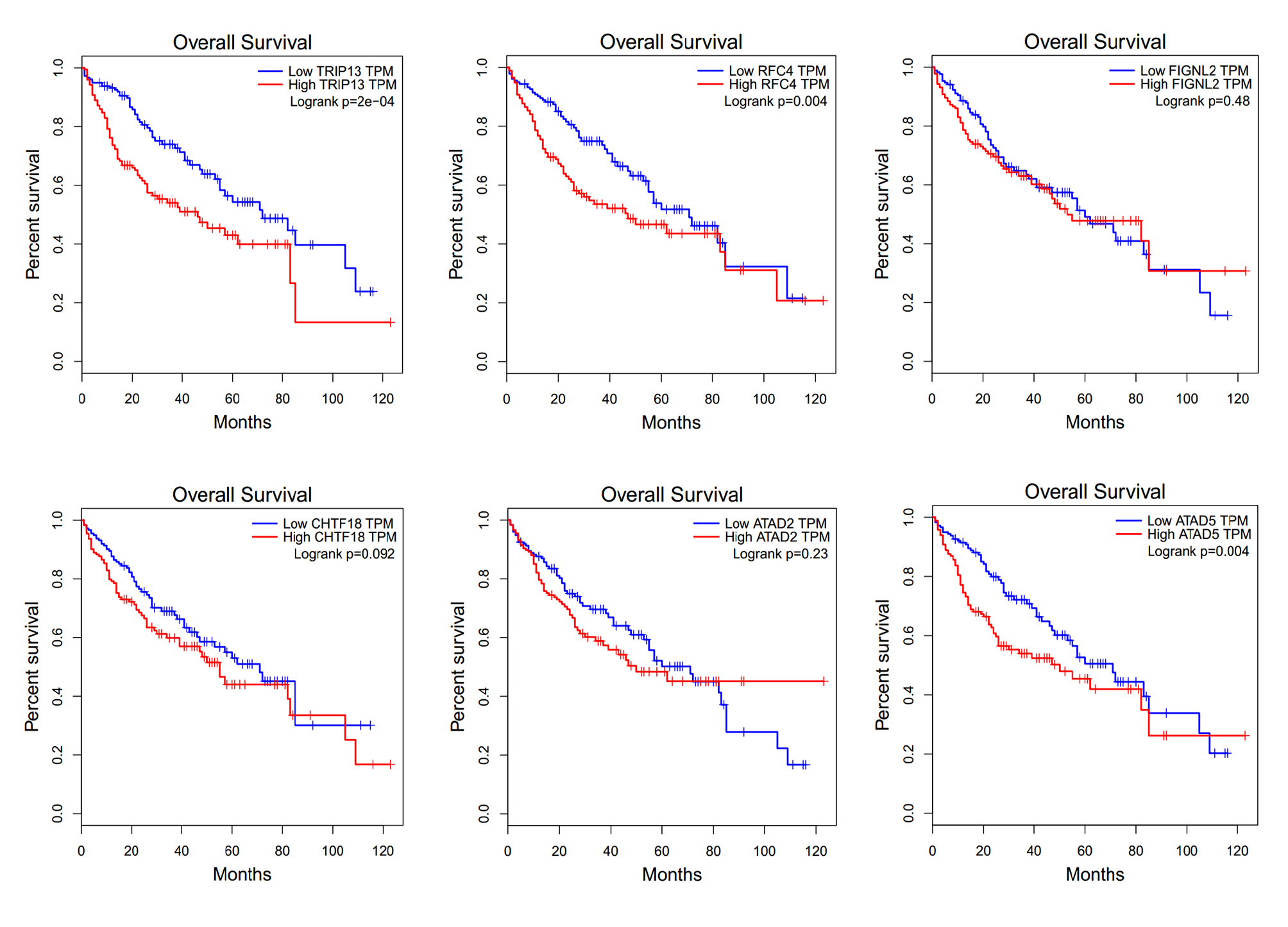

Supplement: Supplementary file 1 — Figure S1. Survival analysis of six differentially expressed AAA ATPase genes. Patients were classified into two groups according to gene expressions. Kaplan–Meier survival curves and log-rank test for different groups were performed. (TIF 9035 kb) [file 13046_2019_1401_MOESM1_ESM.tif]

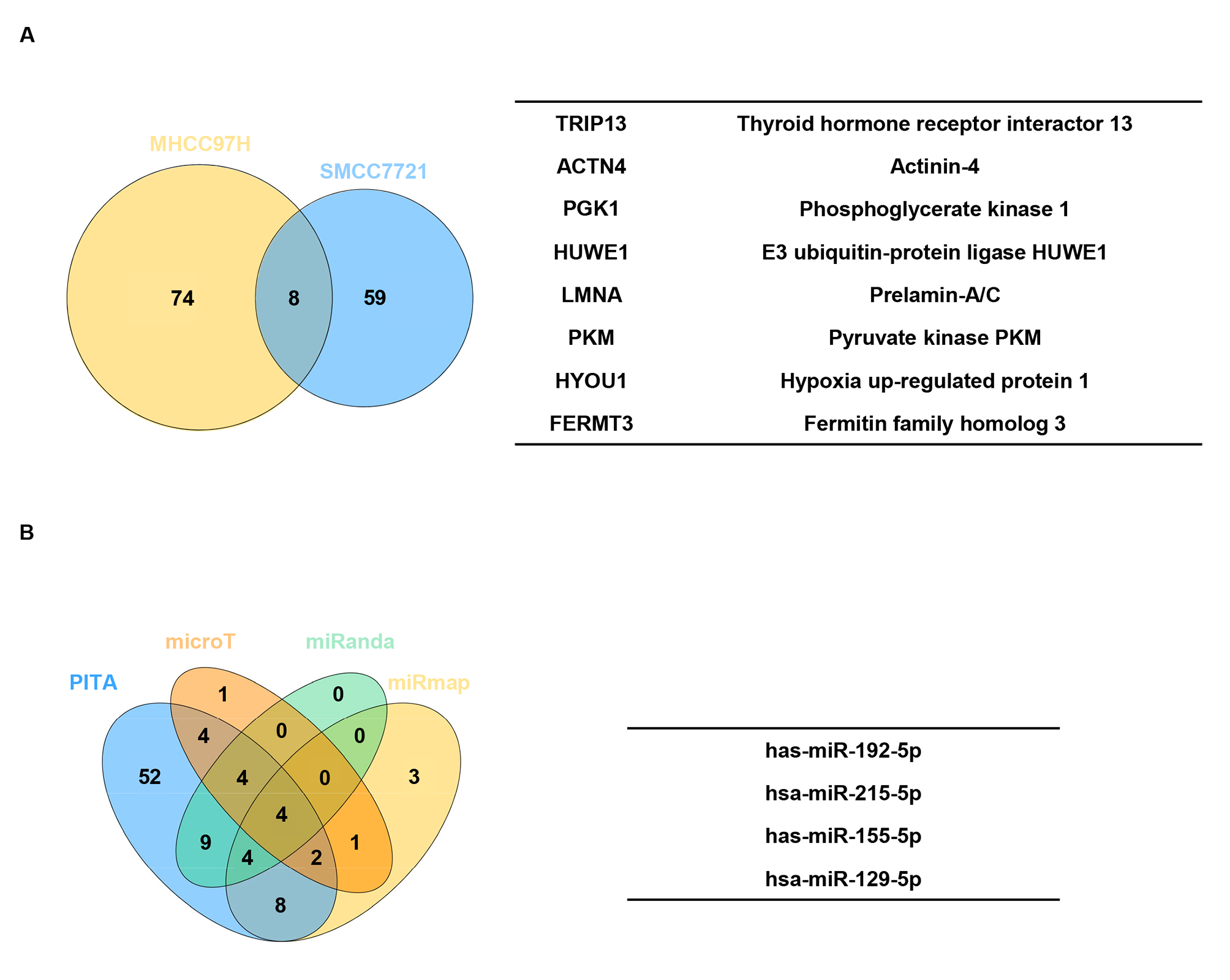

Supplement: Supplementary file 2 — Figure S2. Prediction of TRIP13 binding proteins and microRNAs. A Venn chart showed the number of binding partners of TRIP13 between SMMC7721 and MHCC97H, and 8 overlapped proteins were included in the diagram. Eight overlapped proteins are listed in the Table. B Venn chart showed the number of binding microRNAs of TRIP13 predicted by different databases. Four overlapped microRNAs are listed in the Table. (TIF 9913 kb) [file 13046_2019_1401_MOESM2_ESM.tif]

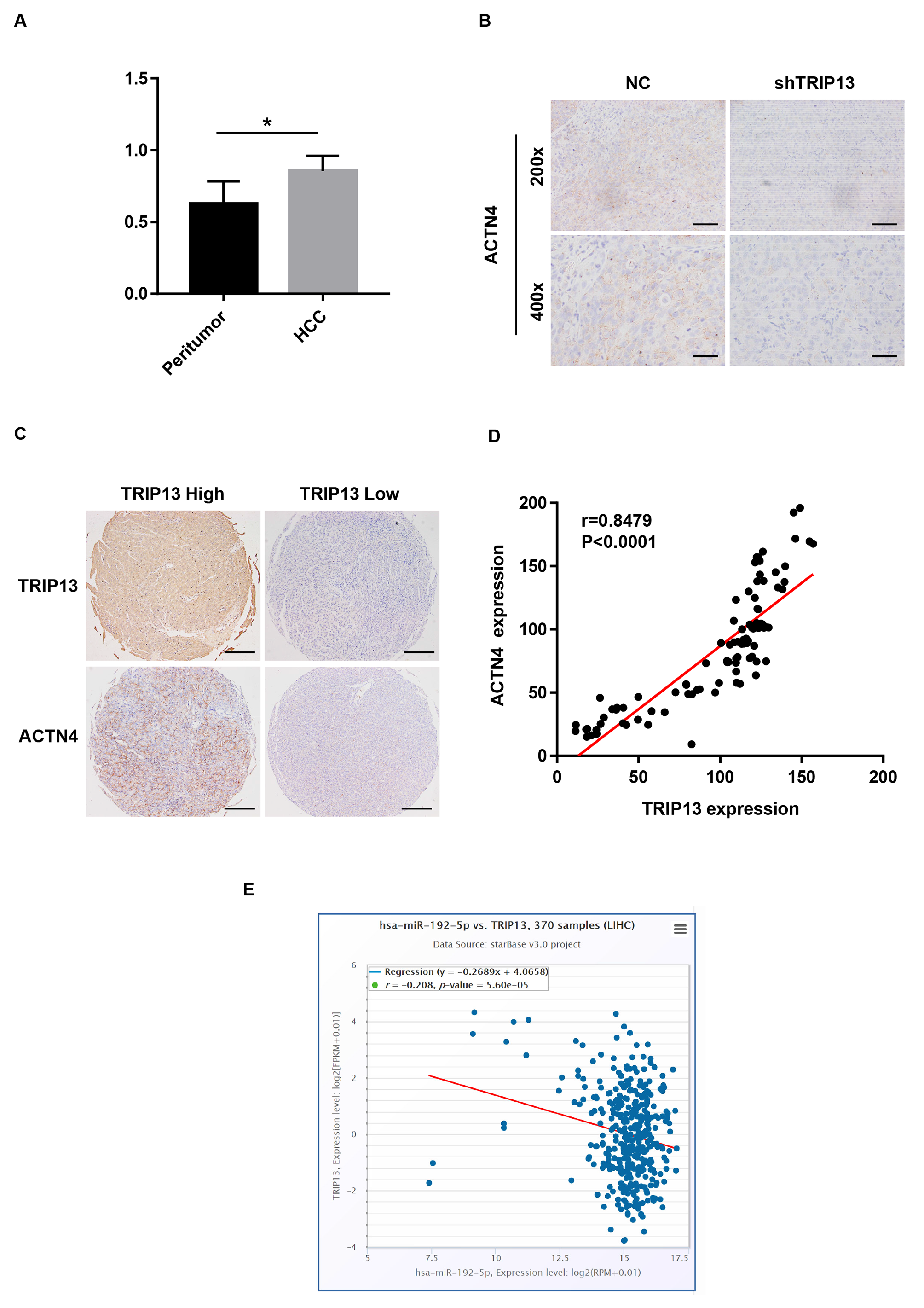

Supplement: Supplementary file 3 — Figure S3. A Statistical analysis of TRIP13 expression in HCC and peritumor samples. B Representative images of ACTN4 IHC in xenograft mouse tumors samples. C-D TRIP13 expression is positively correlated with ACTN4 expression in HCC tissue microarray. E Patients were classified into two groups according to gene expressions. Kaplan–Meier survival curves and log-rank test for different groups were performed. (TIF 20535 kb) [file 13046_2019_1401_MOESM3_ESM.tif]
